# Heterogeneity of mast cells and expression of Annexin A1 protein in a second degree burn model with silver sulfadiazine treatment

**DOI:** 10.1371/journal.pone.0173417

**Published:** 2017-03-09

**Authors:** Helena Ribeiro Souza, Lucas Ribeiro de Azevedo, Lucas Possebon, Sara de Souza Costa, Melina Mizusaki Iyomasa-Pilon, Sonia Maria Oliani, Ana Paula Girol

**Affiliations:** 1 Integrated College Padre Albino Foundation (FIPA), Catanduva, São Paulo, Brazil; 2 Department of Biology, Laboratory of Immunomorphology, São Paulo State University, (UNESP), São José do Rio Preto, São Paulo, Brazil; Queen Mary University of London, UNITED KINGDOM

## Abstract

Mast cells (MCs) participate in all stages of skin healing and one of their mediators is the Annexin A1 protein (AnxA1), linked to inflammation, proliferation, migration and apoptosis processes, but not studied in thermal burns yet. Therefore, our objectives were to evaluate the behavior of MCs and AnxA1 in a second degree burn model, treated or not with silver sulfadiazine 1% (SDP 1%) and associated to macrophages quantification and cytokines dosages. MCs counts showed few cells in the early stages of repair but increased MCs in the final phases in the untreated group. The normal skin presented numerous tryptase-positive MCs that were reduced after burning in all analyzed periods. Differently, few chymase-positive MCs were observed in the early stages of healing, however, increased chymase-positive MCs were found at the final phase in the untreated group. MCs also showed high immunoreactivity for AnxA1 on day 3 in both groups. In the tissue there was a strong protein expression in the early stages of healing, but in the final phases only in the SDP treated animals. TNF-α, IL-1β, IL-6, IL-10 and MCP-1 levels and macrophages quantification were increased in inflammation and reepithelialization phases. Reduced IL-1β, IL-6 and IL-10 levels and numerous macrophages occurred in the treated animals during tissue repair. Our results indicate modulation in the profile of MCs and AnxA1expression during healing by the treatment with SDP 1%, pointing them as targets for therapeutic interventions on skin burns.

## Introduction

The tissue repair process in the healing of burns can be divided into phases of inflammation, proliferation and maturation, where each stage orchestrates the beginning of the next phase [1–4]. The maturation phase is characterized by neogenesis of the epithelial appendages and the extracellular matrix (ECM) remodeling, but pathological scars may be formed due to excessive collagen synthesis [1].

Among the mediators involved in inflammation, there are the pro-inflammatory cytokines interleukin-1 beta (IL-1β), IL-6, tumor necrosis factor-alpha (TNF-α) and the anti-inflammatory cytokine IL-10, which differ in their expression during wound healing [5–7]. The initial phase also involves the release of monocytes chemoattractant protein-1 (MCP-1), that attracts macrophages and mast cells (MCs) [8,9].

The MCs have important functions in the different wound repair phases [10]. In the process of degranulation, MCs release chemotactic factors and specific proteases, such as tryptase and chymase, to the ECM contributing to the degradation of ECM, promotion of angiogenesis and tissue remodeling through selective proteolysis in the matrix and activation of matrix metalloproteinases (MMPs) [11–13]. Previous investigations have also indicated the importance of chymase in the healing process of burn in rats, related to the density of capillaries and accumulation of collagen [12,14]. The variable expression of proteases has led to the recognition of MCs subpopulations in humans and murines [15,16].

Moreover, studies have indicated that the granules of MCs store the protein annexin A1 (AnxA1) [17,18], the first cloned member of the superfamily of annexins proteins. The annexins are grouped according to their structural characteristics and display a central domain consisting of four replicates of 60 to 70 amino acids each, with affinity for Ca^++^ and connected to a N-terminal sequence, which confers specificity of action for each member of the superfamily [19]. The AnxA1 protein presents various functions related to inflammation [20,21] growth [22], migration, cell proliferation and differentiation, besides membrane transport and apoptosis [21,23].

In the skin under normal conditions, the expression of AnxA1 is reduced [24], however, abnormal distribution and expression of the protein have been observed in inflammations and skin tumors [24–27] and mucosal injury [28–30]. The AnxA1 is strongly expressed in skin lesions in lupus [27], in differentiated squamous cell carcinoma [24] and melanoma [26]. In patients infected with *Leishmania braziliensis*, the expression of AnxA1 was stronger in macrophages CD163+ and lymphocytes CD4+ and CD8+ on infected skin compared to normal skin [31]. Immunohistochemical analyzes, in a model of granulomatous inflammation, showed strong expression of AnxA1 in MCs in the initial (7 days) and late (28 days) phases of the inflammatory reaction [25].

The AnxA1 was also indicated as the key mediator in the death of keratinocytes in Stevens-Johnson syndrome and in toxic epidermal necrolysis, important cutaneous drug reactions [32]. The pharmacological treatment with the mimetic peptide of the N-terminal region of AnxA1 (Ac2-26) was used in different models of studies. In vitro investigations indicated that the peptide was able to stimulate the migration of human fibroblast WS1 lineage [33]. In *in vivo* studies, they also showed protective peptide effects with increased skin transplant survival in allograft model in rats [34] and improvement in the healing process of excisions in mice skins, observed in a dose-dependent manner [35]. However, there are no known reports on the expression and function of AnxA1 in the repair of burns.

As MCs and AnxA1 have been little explored in burns, the aim of this study was to analyze the profile of these cells by the assessment of their number and heterogeneity for tryptase and chymase, and also to evaluate the expression of AnxA1 in MCs and in skin flaps in a second degree burn model using the silver sulfadiazine at 1% (SDP 1%), considered the standard treatment for partial thickness burns due to its antimicrobial properties [36,37]. The results of these analyses may be useful for a better understanding of t he role of MCs and the protein AnxA1 in the wound healing process.

## Materials and methods

### Animals

Wistar rats (n = 40) weighing approximately 250g were obtained from the Integrated College Padre Albino Foundation (FIPA). The animals were kept in cages in a controlled environment (24 to 25°C, 12h light/dark cycle) with water and food ad libitum. The experiments were conducted after approval and in accordance with the rules of the Ethics Committee for Animal Use of FIPA (Protocol 12/14).

### Experimental model of burn and treatment protocols

The animals were anesthetized intraperitoneally with 0,2mL/100g of ketamine and 0,05mL/100g of xylazine and submitted to trichotomy of the dorsal region, after a metal block with dimensions of 2x2cm^2^ and water heated to 100°C was applied for 10 seconds to characterize a second degree burn [38]. Immediately after the trauma, the lesions were covered with gauze moistened in cold saline solution. The animals were given analgesic codeine (1mL/kg) right after the injury induction by gavage and it was offered diluted in drinking water on the following days. The control of water intake and weight of the animals was daily performed.

The topical treatments were begun 24h after the induction of burns. The wounds were cleansed with saline daily. Regarding the treatment, two groups were established. One group was the control group and it received no treatment (C Groups), the other group was treated with SDP 1% ointment (SDP Groups) once a day. Each group, control and treated, was subdivided into 4 groups according to the time the lesion was collected, (n = 5/group) so that different phases of the tissue repair could be analyzed. Thus, the animals were euthanized by overdose of isoflurane for the lesions removal after 3, 7, 14 and 21 days of injury. For comparison of the physiological state of the tissue in relation to fragments of regenerated skin, normal skin flaps (n = 5) were also taken from control animals, from not burned regions of skin. The animals’ water and food intake and their behavior in the cages were monitored daily. A veterinarian followed the wounds to check for signs of infection. No animal died as a result of the injuries.

### Quantitative analysis of cytokine levels

Fragments from all groups were macerated in liquid nitrogen and added 650μL of a solution containing protease and phosphatase inhibitors (Merck, Millipore Corporation, USA) following the manufacturer's instructions. The material was incubated for 20 minutes at 4°C under constant agitation and then centrifuged at 14.000 RPM for 10 minutes at 4°C. The supernatants were collected and frozen at -80°C.

TNF-α, IL-1β, IL-6, IL-10 and MCP-1 were quantified in the supernatant using the MILLIPLEX MAP Kit (RECYTMAG-65K; Merck Millipore Corporation, USA) and analyzed on the Luminex xMAP MAGPIX device (Merck Millipore Corporation, USA). The concentration of the analytes was determined by the MAGPIX xPONENT Software and expressed in pg/mL.

### Histopathological analysis and quantification of cells

Fragments of normal skin and lesions were fixed in 4% formalin, processed for inclusion in paraffin and sectioned at 5μm for histopathologic, quantitative and immunohistochemical analysis. The repair process was evaluated histologically by Hematoxylin-Eosin (HE) and the organization of the collagen fibers was evidenced by Picrosirius Polarization method.

MCs were stained with 0.1% toluidine blue (TB+ MCs) and evaluated according to their morphological characteristics in intact or degranulated. The histamine accumulation by MCs was evidenced after staining with 2.5% Safranin-O (S-O+ MCs) [16,39,40]. The quantification of MCs in the skin fragments was performed in 10 images per slide obtained by 40X objective in a Leica microscope (DM500). The areas of each tissue were obtained using the Leica Image Analysis Software.

### Dilutions of the primary antibodies for immunohistochemical analysis

1. Mouse polyclonal anti-AnxA1 (1:700 for the stroma/epithelium within 20 hours of incubation and 1:400 for MCs within 4 hours of incubation; Merck Millipore Corporation, USA).
2. Mouse monoclonal anti-Chymase (1:100; Abcam, USA) and mouse monoclonal anti-Tryptase (1:3000; Merck Millipore Corporation, USA) within 4 hours of incubation.
3. Mouse monoclonal anti-rat monocyte/macrophages (1:150, Merck Millipore Corporation, USA) within 4 hours of incubation.

### Immunohistochemical analysis

Sections of the different samples were prepared on gelatinized slides and then deparaffinized, rehydrated, and after antigen retrieval (citrate buffer pH 6.0 at 96°C for 20 minutes) and blocking of endogenous peroxidase, they were incubated in a humid chamber at 4°C with the primary antibody diluted in 1% BSA. After, the sections were incubated with secondary biotinylated antibody, revealed with DAB substrate and counterstained with Hematoxylin. The negative control reaction was obtained by omitting the primary antibody. The analysis of AnxA1 protein expression in epithelium and stroma from skin fragments was performed by optical densitometry (arbitrary units from 0 to 255) by the program Leica Image Analysis. Densitometric analysis of AnxA1 expression in the cytoplasm of MCs was performed on 10 cells from each sample. The immunoreactive MCs for tryptase (MCT) or chymase (MCC) and macrophages were quantified as previously described.

### Statistical analysis

The results were submitted previously to descriptive analysis and determination of normality then compared by analysis of variance (ANOVA-two way) and Bonferroni post-test. All values were expressed as mean ± S.E.M. and P values less than 0.05 were considered statistically significant.

## Results

### Better healing of the second degree burn lesion after SDP 1% treatment

**Fig 1:**
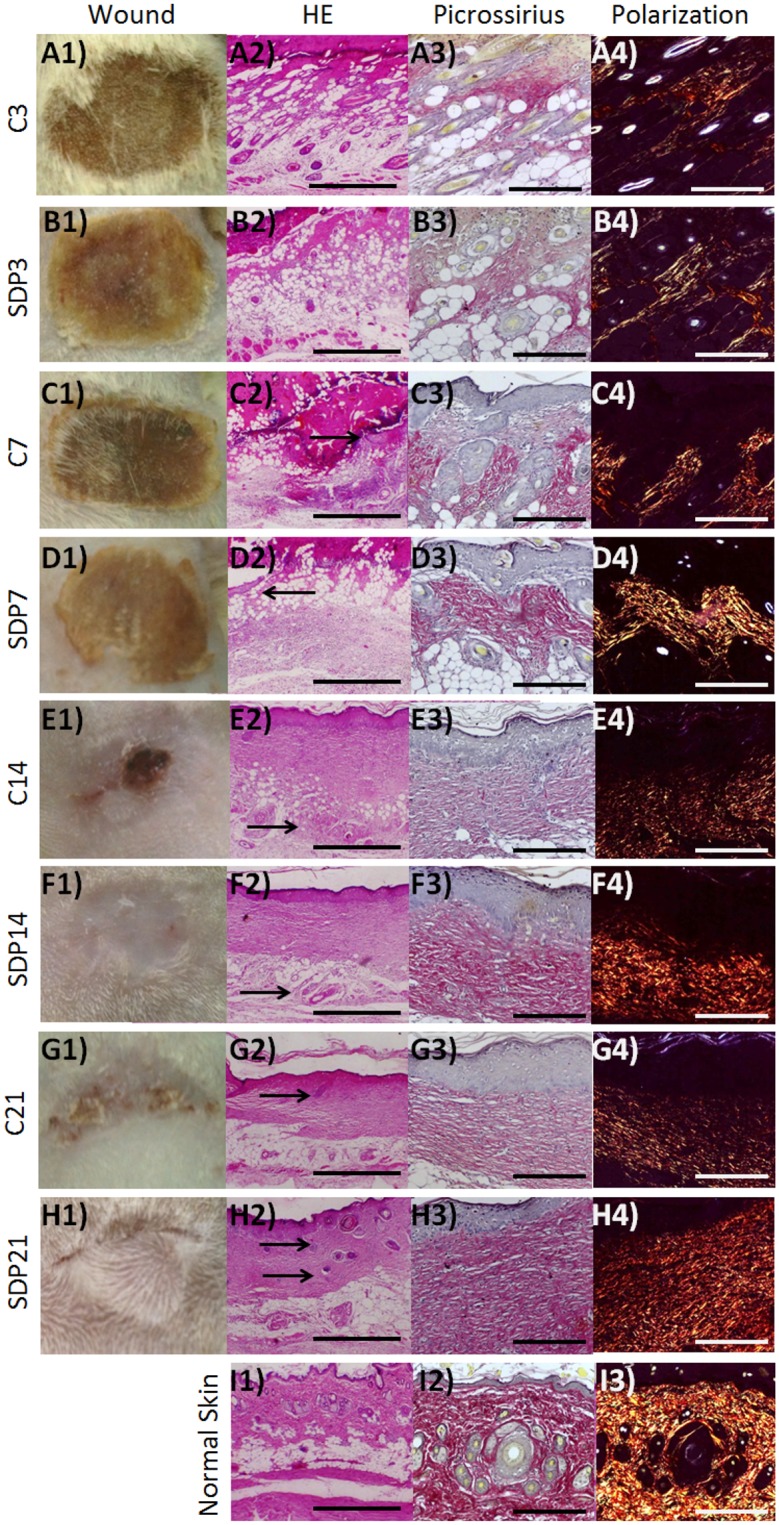
Macroscopic and histopathologic analysis of the healing process in a second degree burn. (a) C3 and (b) SDP3, inflammation phases, with leukocytes influx and presence of adipocytes in both groups. (c) C7 and (d) SDP7, proliferation phase with re-epithelialization (arrows). Up to 7 days weakly stained collagen fibers (a3, b3, c3 and d3) and low birefringent after polarization (a4, b4, c4 and d4) in the dermis of both groups. (e) C14 and (f) SDP14, complete reepithelialization and fast healing in SDP 1% group (e1 and f1). Dermis and hypodermis (e2, f2—arrows) are better organized and collagen fibers more strongly stained in the group treated with SDP 1% (e3, f3). (g) C21 and (h) SDP21, remodeling phase, the epithelial attachments (g1, h1, g2, h2—arrows) may be observed in larger amount and with increased birefringence under polarized light in the groups treated with SDP 1% (g4 and h4). Normal Skin (i). (a1, b1, c1, d1, e1, f1, g1, h1 and i1) Macroscopic analisys. (a2, b2, c2, d2, e2, f2, g2, h2 and i2) Staining: HE. Bars 500 μm. Picrossirius staining without (a3, b3, c3, d3, e3, f3, g3, h3 and i3) and after (a4, b4, c4, d4, e4, f4, g4, h4 and i4) polarization. Bars 200μm.

The histopathological analysis confirmed the characteristics of second degree burns (Fig 1A2 and 1B2) and, together with the macroscopic analysis, showed the best evolution of the wounds in the SDP groups (Fig 1F and 1H). During the wound healing process, 3 days after the burn induction, common characteristics of the inflammation phase, as the influx of leukocytes and adipocytes proliferation, were observed (Fig 1A2 and 1B2). Seven days later, a reepithelialization process was verified, featuring the cellular proliferation phase (Fig 1C2 and 1D2), whereas 14 days post injury (Fig 1E and 1F), the proliferation of the ECM was evident. On day 21 (Fig 1G and 1H) the remodeling phase was characterized with formation of epithelial appendages, epidermis and dermis restructuring and changes in the patterns of birefringence of collagen fibers, observed after polarization, indicating reorganization, especially in the skin of the animals treated with SDP 1% (Fig 1H).

### Treatment with SDP 1% reduces cytokines in the late stage of healing and increases amount of macrophages in the stage of cell proliferation

**Fig 2:**
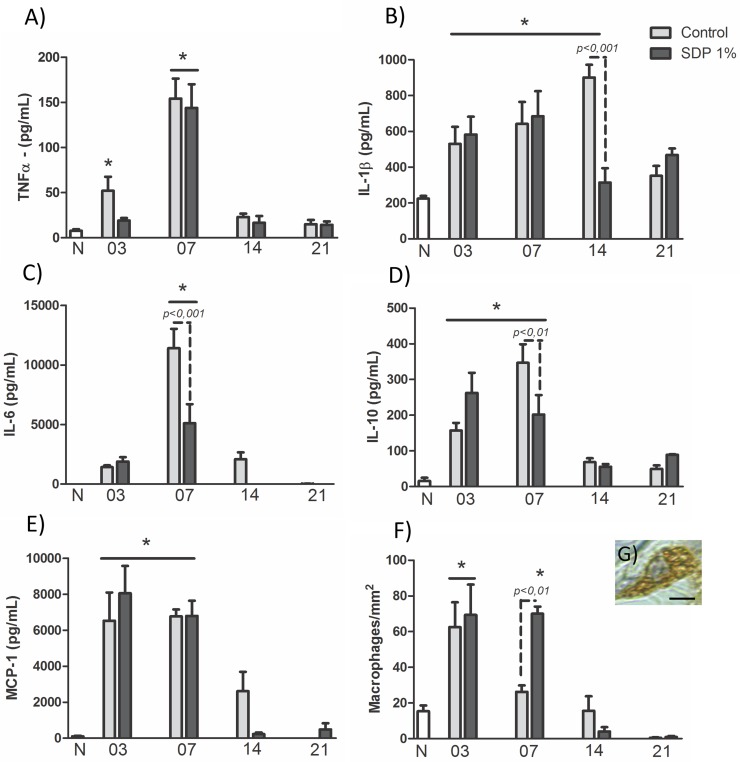
Cytokines and macrophages in wound healing in a second degree burn. (a) TNF-α: high dosages in C3 and in both groups after 7 days. (b) IL-1β: high levels in both groups on days 3 and 7 and also in C14. (c) IL-6: overexpression on day 7, especially in the control group. (d) IL-10: Increased levels on days 3 and 7. (e) MCP-1: overexpression on days 3 and 7. (f) Macrophages: numerous macrophages on day 3 and SDP7. (g): Macrophage. Counter-staining: Hematoxilin. Bar 2μm. Values are presented as mean ± S.E.M. (n = 5/group). * *p<0*.*05 vs* Normal Skin (N).

The TNF-α quantification showed after 3 days of injury a significant increase (*p<0*.*05*) only in C3 group compared to N (N: 7.78 ± 2.29; C3: 52.13 ± 18.92; Fig 2A) while on day 7 higher TNF-α levels were found in both groups compared to N (*p<0*.*001*) and also to previous phase (C7: 154.18 ± 27.14, *p<0*.*001 vs* C3; SDP7: 143.98 ± 31.91, *p<0*.*001 vs* SDP3; Fig 2A).

The IL-1β dosages indicated a significant increase in both groups, on days 3 and 7 compared to N group (N: 224.22 ± 21.21; C3: 530.64 ± 94.37; *p<0*.*05*; SDP3: 582.94 ± 99.01; *p<0*.*01*; C7: 641.79 ± 122.76; *p<0*.*01*; SDP7: 684.68 ± 140.04; *p<0*.*001*; Fig 2B). Interestingly, in the next period, there was a higher amount of this cytokine in the C14 group in relation to N (*p<0*.*001*) and reduction of these levels in SDP14 group compared to the previous phase (*p<0*.*05*) and the control group (C14: 901.29 ± 70.61; SDP14: 314.39 ± 79.83, *p<0*.*001 vs* C14; Fig 2B).

Regarding the IL-6, overexpression (*p<0*.*001*) of this cytokine was observed in the lesions collected on day 7 compared to N (N: 0.49 ± 0.02; C7: 9192.64 ± 2743.42; SDP7: 5100.97 ± 1600.30; Fig 2C), with lower dosage in SDP7 (*p<0*.*001*) compared to C7 group.

The quantification of IL-10 showed higher levels in the inflammation (N: 15.67 ± 12.76, C3: 157.19 ± 21.29, *p<0*.*05*; SDP3: 262.90 ± 68.59; *p<0*.*001*; Fig 2D) and cell proliferation phases (C7: 347.06 ± 63.59; *p<0*.*001*; SDP7: 202.02 ± 66.58; *p<0*.*01*; Fig 2D), in relation to N. Again, the treatment with SDP 1% reduced the cytokine levels on day 7 compared to control (*p<0*.*01*).

Finally, the dosages of the MCP-1 chemokine showed elevated levels (*p<0*.*001*) on days 3 and 7 of the healing process in relation to N (N: 100.27 ± 50.95; C3: 6525.44 ± 1569.07; SDP3: 8051.84 ± 1520.58; C7: 6774.98 ± 467.06; SDP7: 6796.24 ± 845.05; Fig 2E). Similarly, in the quantification of macrophages (Fig 2G), there was an increase (*p<0*.*001*) in the number of these cells in both groups 3 days after the injury compared to N (N: 16.15 ± 4.43; C3: 60 ± 19.41; SDP3: 75 ± 22.68; Fig 2F). However, in the next phase, only the SDP7 group presented numerous macrophages compared to N (SDP7 70 ± 5.77; *p<0*.*001*; Fig 2F).

### The number and morphology of the MCs are modulated by treatment with SDP 1%

**Fig 3:**
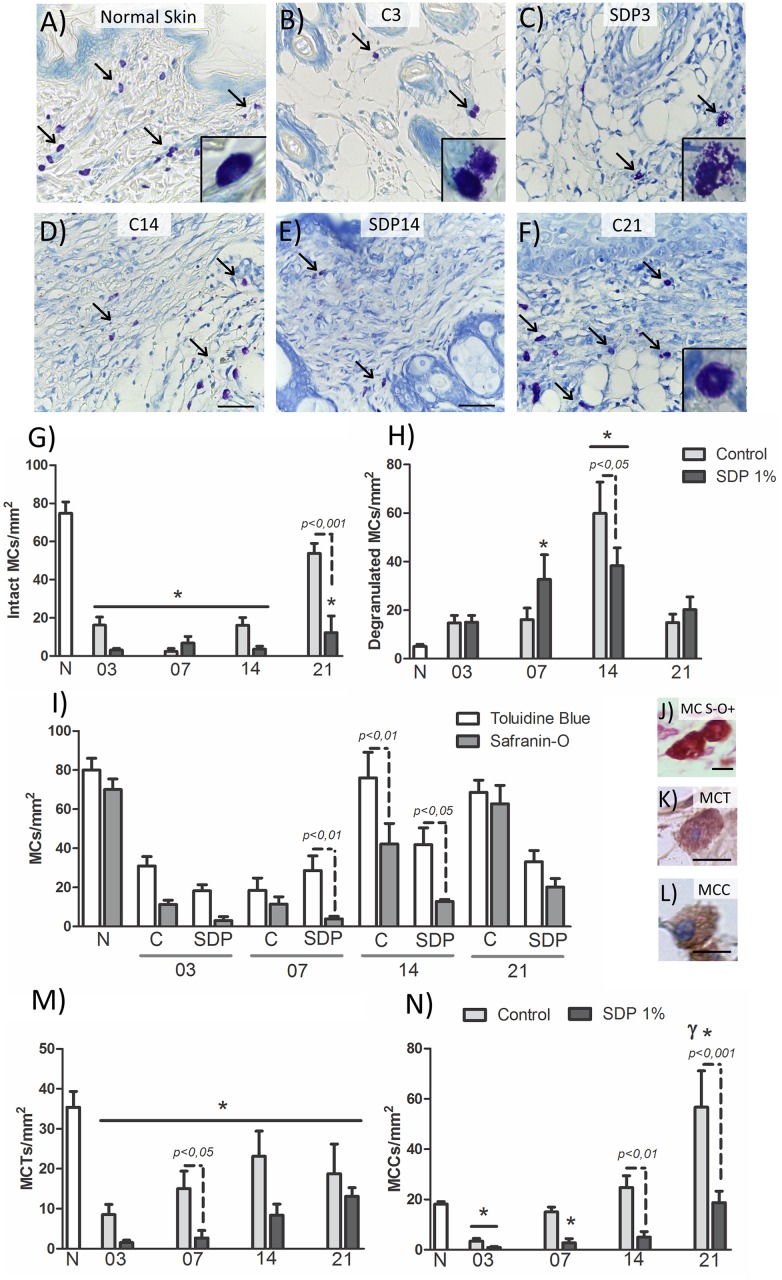
Morphology of MCs in burn healing process. (a) MCs (arrows), most intact in Normal Skin (N). (b) C3 and (c) SDP3 with few MCs and mostly degranulated. (d) C14 (e) SDP14, MCs degranulated, mainly in control group. (f) C21, numerous intact MCs. Details of intact (a and f) and degranulated MCs (b and c). Staining: Toluidine Blue. Bars 50 μm. Quantification of MCs: (g) numerous intact MCs in N and C21 and (h) degranulated MCs in SDP7 and in both groups on day 14. (i) Differences between quantification of MCs evidenced by Toluidine blue or Safranin-O, with few MCs S-O+ (MCs histamine storage) in SDP7, C14 and SDP14 compared to the MC TB+ (total MCs in the tissue). (j) MC stained with Safranin-O (MC S-O+); (k) reactive MC for tryptase (MCT) and (l) chymase (MCC); Bars 50 μm. Heterogeneity of MCs, showing many MCTs (m) in N and numerous MCCs (n) in C21. Values are presented as mean ± S.E.M. (n = 5/group). * *p<0*.*05 vs* N and γ *p<0*.*001 vs* C14.

After confirming the lesion model, we quantified the MCs in the region of the lesion as evidenced by the Toluidine Blue dye and morphologically differentiate these cells between intact mast cells, that is, cells with well-defined contour and that are not in the clear process of releasing the contents of their cytoplasmic granules (Fig 3A and 3F), and degranulated MCs with irregular contours and dispersed granules (Fig 3B and 3C). Quantification of MCs showed a large number of intact cells in N (N: 74.91 ± 5.84; Fig 3A and 3G). On days 3 and 7, in both groups, there was a significant decrease in the total number of MCs compared to N (C3: 30.94 ± 4.89; SDP3: 18.28 ± 3.08; *p<0*.*001*: 80.01 ± 6.01; C7: 18.51 ± 8.12; SDP7: 39.55 ± 13.29 *p<0*.*01*; Fig 3B, 3C and 3G). In the SDP group, the MCs were observed mostly degranulated 7 days post injury (SDP7: 32.73 ± 22.55, *p<0*.*05 vs* N: 5.09 ± 1.97; Fig 3H).

In the final phases of repair, the amount of MCs increased only in C group. These cells were observed mostly degranulated 14 days post injury (C14: 59.88 ± 12.87 *p<0*.*05 vs* SDP14: 38.31 ± 9.55; Fig 3D and 3H) but intact after 21 days (C21: 53.75 ± 6.81 *p<0*.*001 vs* SDP21: 15.66 ± 5.66; Fig 3F and 3H).

### Histamine storage by mast cells during healing

After observing the modulation promoted by SDP 1% in MCs, reducing their amount in the final stages of repair, as well as the number of degranulated MCs, we analyzed the histamine accumulation in the citoplasmatic granules of MCs, during the tissue repair by reaction to S-O (Fig 3J) [(40)]. Then, the quantification of the TB+ MCs (Total MCs) was compared to the S-O+ MCs (MCs with accumulation of histamine) (Fig 3I).

Numerous S-O+ MCs were observed in N (S-O+: 70 ± 7.01; Fig 3I). In the lesions, a marked difference between TB+ MCs and S-O+ MCs occurred on day 7 only in the SDP group (SDP7 TB+: 39.55 ± 29.73 *p<0*.*01 vs* SDP7 S-O+: 3.94 ± 1.28; Fig 3I) and on day 14 in both groups (C14 TB+: 76 ± 13.14 *p<0*.*01 vs* C14 S-O+: 42.17 ± 10.51; SDP14 TB+: 41.9 ± 11.01 *p<0*.*05 vs* SDP14 S-O+: 20.83 ± 5; Fig 3I). This difference between TB+ MCs and S-O+ MCs occurred in the same periods in which these cells were in degranulation process. It indicates little accumulation of histamine within MCs in these periods, possibly due to degranulation.

### Treatment with the SDP 1% influences the heterogeneity of MCs

To analyze the heterogeneity of MCs during the healing process these cells were quantified after immunohistochemistry for the tryptase (MCT; Fig 2K) and chymase (MCC; Fig 2L). In the N group, the MCs were mostly tryptase + (MCT: 33.3 ± 4.79; Fig 3M MCC: 18.7 ± 2.1; Fig 3N).

At all stages of tissue repair a reduction of MCTs was observed in both groups compared to N, with greater differences (*p<0*.*001*) in the early stages (C3: 8.5 ± 2.57; SDP3: 1.5 ± 0.61; C7: 15 ± 5.68; SDP7: 2.5 ± 1.93; Fig 3M) than in the final stages of repair (C14: 18.65 ± 7.81; *p<0*.*05 vs* N; SDP14: 10.5 ± 4.56; *p<0*.*01 vs* N; C21: 18.75 ± 9.60; *p<0*,*05 vs* N; SDP21: 13.12 ± 2.77; *p<0*.*01 vs* N; Fig 3M).

On day 3 of the healing, there was a reduction in the number of MCCs (*p<0*.*001*), compared to N group (C3: 4 ± 1.27; SDP3: 0.87 ± 0.52; Fig 3N), however, after 7 days of injury, the amount of MCCs was reduced (*p<0*.*05*) only in SDP7 compared to N group (C7: 12.5 ± 4.67; SDP7: 2.8 ± 1.64; Fig 3N). On day 14, the treated group showed fewer MCCs (*p<0*.*01*) compared to C14 (C14: 24.75 ± 5.37; SDP14: 5 ± 2.23; Fig 3N). Finally, after 21 days of burn lesion, increased MCCs were found in C21 group in relation to the other groups (C21: 60 ± 21.21, *p<0*.*001 vs* N and *p<0*.*01 vs* C14; SDP21: 18.75 ± 5.90, *p<0*.*001 vs* C21; Fig 3N).

### The expression of AnxA1 in the cytoplasm of MCs, epidermis and dermis varies during the wound healing

**Fig 4:**
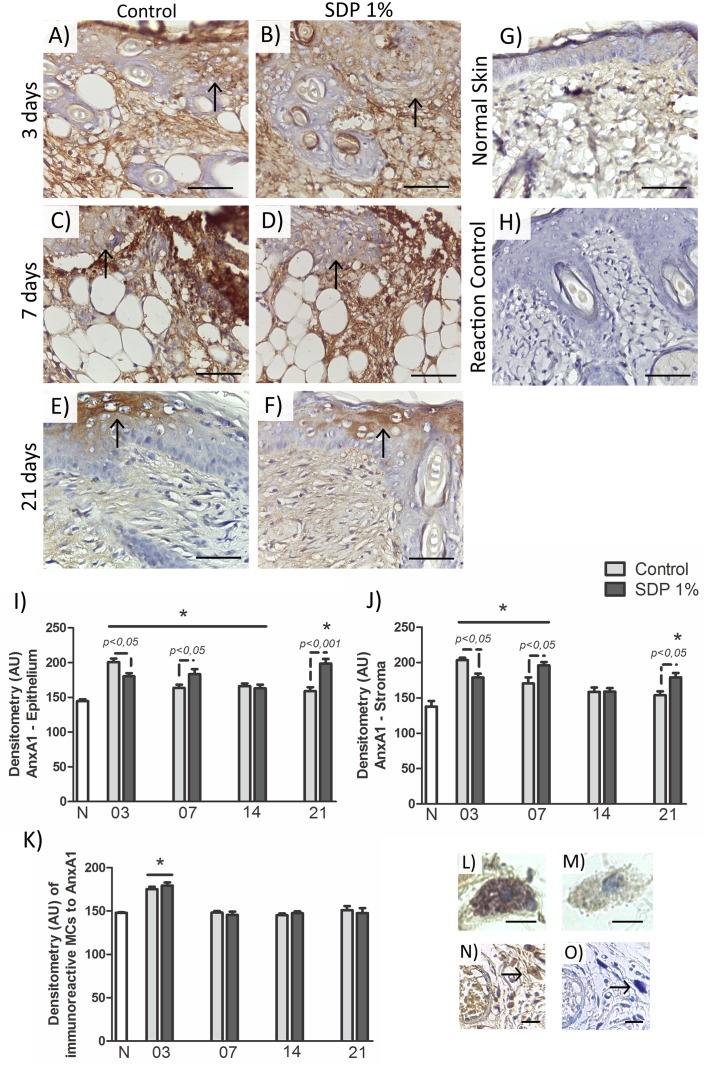
AnxA1 expression in the second degree burn healing process. (a) C3 and (b) SDP3, increased expression of the protein in the inflammation phase, 3 days post injury, in the surface epithelium (arrows) and dermis. (c) C7 and (d) SDP7, increased expression in the stroma and epithelium (arrows), 7 days post injury. (e) C21 and (f) SDP21 higher expression of AnxA1 in the group treated with SDP 1%, surface epithelium (arrows). (g) Low expression of AnxA1 in normal skin. (i) No immunoreactivity in the reaction control. Counter-staining: hematoxylin. Bars 50μm. Optical densitometry of immunostained AnxA1 in (i) Epithelium and (j) Stroma. (k) Densitometric analysis of AnxA1 in the cytoplasm of MCs. Values are presented as mean ± S.E.M. (n = 5/group). Values *p<0*.*05 vs* Normal Skin (N). (l) strongly AnxA1 immunoreactive MC in the inflammatory phase. (m) weakly AnxA1 immunoreactive MC, remodeling phase. Bars 20 μm. (n) MC immunoreactive for AnxA1 and (o) the same cell in serial seccion with counterstaining of hematoxylin and toluidine blue, arrows indicate MCs. Bars 50 μm.

A reactivity for the AnxA1 protein was observed in the cytoplasm of MCs (Fig 4L and 4M). The densitometric analysis showed increased immunostaining for AnxA1 (*p<0*.*001*) in the phase of inflammation, in C3 and SDP3 groups compared to the others (Fig 4K). The specificity of the immunolabeling was checked by control of the reaction and the counterstaining with TB in serial sections.

In the tissues, there was reduced AnxA1 protein expression in the normal skin (Fig 4G, 4I and 4J). After burn, on day 3 (Fig 4A and 4B), the expression of AnxA1 was increased (*p<0*.*001*) in both groups in the epithelium, especially in keratinocytes (Fig 4I) and stroma (Fig 4J) compared to N, especially in the C3 group (*p<0*.*05*; Fig 4I and 4J).

On day 7, the AnxA1 expression remained increased with respect to N (Fig 4C and 4D), in regions of reepithelization (C7: *p<0*.*05*; SDP7: *p<0*.*001*; Fig 4I) and in the dermis (*p<0*.*001*; Fig 4J). However, the SDP7 group showed higher immunostaining for AnxA1 compared to C7 group (*p<0*.*05*; Fig 4I and 4J).

After 14 days of injury, the immunoreaction for AnxA1 was high only in the epithelium in C (*p<0*.*01*) and SDP groups (*p<0*.*05*) compared to N (Fig 4I). Thereafter, further increase of the protein expression occurred in SDP21 group (Fig 4F) compared to N and C21 groups (Fig 4E) in the dermis (SDP21: *p<0*.*001 vs* N; *p<0*.*05 vs* C21; Fig 4J) and epidermis (*p<0*.*001 vs* N and C21; Fig 4I) especially observed near the epithelial appendages development.

## Discussion

Burns are complex traumas, with high morbidity and mortality rates [41,42] and generate strong economic and psychosocial impact due to treatment time [43].

In burn lesions, the interactions between inflammatory cells, chemical mediators, growth factors and ECM, coordinate the healing process, and angiogenesis is essential for tissue remodeling [4,44]. The MCs and the anti-inflammatory protein AnxA1 are important in the wound repair and angiogenesis processes [10,34,35,45]. However, the biological roles of MCs and AnxA1 are still poorly explored in burns. For these reasons, in the present study we investigated the heterogeneity of MCs and the expression of AnxA1 during the healing process of second degree lesions in control animals and animals treated with SDP 1%.

Histopathological findings confirmed the depth of the injury as a superficial second degree burn [38] and also the best regeneration in the treated group [36,46]. Furthermore, on day 21 of the repair process, the analysis of the lesions showed formation of epithelial appendages, especially in the SDP group. The appendages are functional parts of the skin, therefore, a fast regeneration is important to the restoration of normal anatomy, structure and function [4].

Next, knowing the importance of the inflammatory mediators, levels of TNF-α, IL-1β, IL-6, IL-10 and MCP-1 were quantified in the supernatant of the macerated fragments. On days 3 and 7, the dosages of IL-1β, IL-10 and MCP-1 were increased compared to normal skin. Differently, the higher levels of IL-6 were observed only 7 days post injury. The TNF-α dosages were elevated in both burned groups on day 7, but only in the control group on day 3. Increased levels of IL-1β were also found in the control group 14 days after injury. The results of our measurements are consistent with other studies in skin burns [5–9,47].

The pro-inflammatory cytokines TNF-α, IL-1β and IL-6 and the chemokine MCP-1 are important for leukocyte recruitment and reepithelialization [8]; nevertheless, the prolonged inflammation can be harmful [36]. However, our evaluations showed that the treatment with SDP 1% decreased levels of IL-6 and IL-1β and increased the dosage of MCP-1 on day 7. Besides, the macrophage quantification followed the cytokines levels. In an *in vitro* study, the dosage of MCP-1 was decreased in fibroblasts derived from keloids, emphasizing the protective role of MCP-1 in the healing process [48]. This chemokine is released primarily by keratinocytes near the injury and recruits monocytes, macrophages, lymphocytes and also MCs [8,9].

After verifying that the model used in this study was suitable for the induction of second degree burns, we proceed to the analysis of MCs. The MCs were observed in large quantities and mainly intact in the dermis of the normal skin, with a significant reduction of total number on days 3 and 7 after injury, as in other investigations [3,12,14]. In addition, mice deficient in MCs presented an impaired skin wound closure, indicating the importance of the mediators of these cells to this process [49].

Later, in the following phases, there was an increase of MCs number in the untreated group, as also observed in other studies [12,14]. The MCs were found especially degranulated on day 14, but intact 21 days after injury in the control group. The reduction of these cells in the treated group may be related to the leukopenic property of SDP, due to cytotoxicity promoted by the drug components to stem cells from bone marrow [36,50].

Several *in vivo* and *in vitro* studies have linked the increased presence of MCs with the proliferation of fibroblasts and formation of keloids [51–55]. Thus, the control of MCs proliferation in the repair of the skin lesion is an important therapeutic strategy in the treatment of burn patients. Still, in the later stages of healing, a decrease in the number of macrophages and an increase in the number of MCs [3] were observed, characteristics which were also found in our cell quantifications.

With that in mind, following the investigation, we studied the histamine accumulation and heterogeneity of MCs. The process of histamine accumulation was verified by S-O+ MCs, comparing these cells to TB+ MCs. Our results showed differences in the number of TB+ and S-O+ MCs in the same periods in which these cells were found to be active, corroborating that the MCs were still releasing the content of their granules in these periods.

Regarding the heterogeneity of MCs for tryptase and chymase, after 3 days of burn, decreased immunoreactive cells were observed for both proteases. The reduction in the number of MCTs remained in all examined periods of wound healing. These results are in agreement to another study [56] that showed the reduction of MCTs and MCCs in human skin recent wounds. The same study found increased tryptase activity in older scars when compared to normal human skin. In addition, tryptase is considered a biomarker for mastocytosis [57]. Given the MCCs, our analysis showed reduced cells in the initial phases, but numerous MCCs were observed in the lesions from day 14. In the lesions of the untreated groups, the increased MCCs in the late phases of repair match with the elevated IL-1β levels found in our quantifications and are in line with studies that indicate the involvement of chymase in the cleavage of pro-IL-1β to IL-1β, activating it [58]. Furthermore, these results indicate the relation between the MC histamine storage and chymase accumulation and are in agreement with a study that demonstrated the importance of histamine to the maturation of MCs and accumulation of proteases [40]. Additionally, other *in vitro* study demonstrated that during MC maturation process, the chymase is expressed by most cells and in larger quantities than tryptase [59], indicating again that, in our model, these cells are in the process of infiltration and maturation in the newly regenerated tissue.

Besides that, several studies associate the chymase to the formation of fibrosis and keloids [10,52,53,60,61]. While other investigations indicate that both tryptase and chymase are important for collagen degradation [13,49,56,62]. Thus, our results reinforce that the heterogeneity of MCs, in different periods and in response to treatment with SDP 1%, is related to their function in the cellular microenvironment, indicating that the MCs actively participate in the remodeling phase of the repair process.

Continuing our study, we evaluated the expression of AnxA1 in the MCs cytoplasm, during tissue healing and normal skin. Our evaluations showed strong MC immunoreactivity for the protein, mainly on day 3 in the inflammatory phase in both groups. These results are in accordance to different investigations that also showed the presence of AnxA1 in the granules of MCs [17–19,63] with increased synthesis during the inflammatory processes [63].

Due to the importance of AnxA1, demonstrated by the pharmacological treatment with the mimetic peptide Ac2-26 in a skin allograft model in rats that increased the survival of the transplantation related to inhibition of neutrophil transmigration and induction of apoptosis, reducing tissue damage, [34] and also by the application of hydrogels containing the AnxA1 peptide that provided rapid healing of dorsal wounds in mice [35], we analyzed the expression of Anxa1 in the epidermis, especially in keratinocytes, and in the dermis during the healing process on burns. In the tissue evaluations, a weak immunostaining was found in normal skin, in accordance with previous studies [24,64]. However, an increased AnxA1 expression was observed in both groups 3 and 7 days after injury, which was observed strongly expressed in the dermis and in reepithelialization. These results relate the expression of AnxA1 to the inflammatory process, corroborating with other researches in different models of inflammation [20,65], and are also in agreement with studies that have shown the participation of AnxA1 in the process of cellular proliferation [21] and epidermal proliferation [24]. Other studies have indicated the importance of AnxA1 in different pathological processes, such as in inflammations and neoplasias [26,64], in the intestinal mucosa during the closure of intestinal epithelium lesions [28–30], migration and proliferation of endothelial cells [22], differentiation of keratinocytes [24] and their stratification and keratinization [66] and also in the motility of fibroblasts [67].

In the remodeling phase, on day 21, higher immunostaining for AnxA1 was observed in the SDP group, both in the stroma and epithelial regions, especially close to the neogenesis of hair follicles and glands. The presence of AnxA1 was detected in the duct of sweat glands, demonstrated by in situ hybridization, in another research [64]. In addition, studies have contributed to the understanding of the AnxA1 antifibrotic properties in the matrix of different organs, by decreasing inflammation and subsequent development of fibrosis [68] or by acting directly on the phenotype of fibroblasts, reducing the expression of collagen, α-smooth muscle actin (α-SMA) and the growth transformation factor (TGF-β1) [69,70]. Therefore, our results indicate the role of AnxA1 in tissue repair in burns, both in epithelial regeneration as in the protection against keloid formation.

Moreover, IL-6 and TNF-α can be linked to AnxA1 expression [71]. Although IL-6 can induce the expression of AnxA1 [19], in turn, the AnxA1 can equally reduce TNF-α and IL-6 expressions [71], which explains the modulation of these cytokines during the repair process after 7 days of injury and in the SDP group, when the AnxA1 expression was increased. Our observations also indicated increase in IL-10 levels in the phases of inflammation and cell proliferation, that is again coincident with the phases in which the AnxA1 protein expression is higher in the lesions. While pro-inflammatory cytokines perpetuate inflammation, other mechanisms operate to regulate this activity, such as IL-10 [8] and AnxA1 protein [21,23]. Thus, our results suggest that AnxA1 protein and IL-10 act together in the burn healing process.

In general, our investigation revealed that degranulation of MCs in the early phases of wound healing is important for modulation of the microenvironment with the secretion of proteases and the AnxA1 protein, which may stimulate the release of chemical mediators such as TNF-α, IL-1β, IL-6, IL-10 and MCP-1. Furthermore, our analysis showed that treatment with SDP 1% promotes slower repopulation of the tissue by MCs, controlling mainly MCCs, which may exert a protective effect against the formation of keloids. In addition, our studies revealed increased expression of the protein AnxA1 especially in the treated group, concomitant with keratinocytes proliferation and differentiation and also stromal remodeling, with the possibility of anti-fibrotic action.

Altogether, our data show modulation in the number, degranulation state, maturity and heterogeneity of MCs, and the relation of these cells with the AnxA1 and cytokines expression during the tissue repair process in a model of second degree burn. However, additional studies are needed to better understand the biological role of MCs and their mediators, the AnxA1 protein among them, during the phases of skin repair in burns in the search for new therapeutic tools.
